# Skeletal Muscle DNA Methylation and mRNA Responses to a Bout of Higher versus Lower Load Resistance Exercise in Previously Trained Men

**DOI:** 10.3390/cells12020263

**Published:** 2023-01-09

**Authors:** Casey L. Sexton, Joshua S. Godwin, Mason C. McIntosh, Bradley A. Ruple, Shelby C. Osburn, Blake R. Hollingsworth, Nicholas J. Kontos, Philip J. Agostinelli, Andreas N. Kavazis, Tim N. Ziegenfuss, Hector L. Lopez, Ryan Smith, Kaelin C. Young, Varun B. Dwaraka, Andrew D. Frugé, Christopher B. Mobley, Adam P. Sharples, Michael D. Roberts

**Affiliations:** 1 School of Kinesiology, Auburn University, Auburn, AL 36849, USA; 2 The Center for Applied Health Sciences, Canfield, OH 44406, USA; 3 TruDiagnostic, Lexington, KY 40503, USA; 4 Edward Via College of Osteopathic Medicine, Auburn, AL 24060, USA; 5 College of Nursing, Auburn University, Auburn, AL 36849, USA; 6 Institute for Physical Performance, Norwegian School of Sport Sciences, 0863 Oslo, Norway

**Keywords:** resistance exercise, training volume, DNA methylation, transcriptomics

## Abstract

We sought to determine the skeletal muscle genome-wide DNA methylation and mRNA responses to one bout of lower load (LL) versus higher load (HL) resistance exercise. Trained college-aged males (*n* = 11, 23 ± 4 years old, 4 ± 3 years self-reported training) performed LL or HL bouts to failure separated by one week. The HL bout (i.e., 80 Fail) consisted of four sets of back squats and four sets of leg extensions to failure using 80% of participants estimated one-repetition maximum (i.e., est. 1-RM). The LL bout (i.e., 30 Fail) implemented the same paradigm with 30% of est. 1-RM. Vastus lateralis muscle biopsies were collected before, 3 h, and 6 h after each bout. Muscle DNA and RNA were batch-isolated and analyzed using the 850k Illumina MethylationEPIC array and Clariom S mRNA microarray, respectively. Performed repetitions were significantly greater during the 30 Fail versus 80 Fail (*p* < 0.001), although total training volume (sets × reps × load) was not significantly different between bouts (*p* = 0.571). Regardless of bout, more CpG site methylation changes were observed at 3 h versus 6 h post exercise (239,951 versus 12,419, respectively; *p* < 0.01), and nuclear global ten-eleven translocation (TET) activity, but not global DNA methyltransferase activity, increased 3 h and 6 h following exercise regardless of bout. The percentage of genes significantly altered at the mRNA level that demonstrated opposite DNA methylation patterns was greater 3 h versus 6 h following exercise (~75% versus ~15%, respectively). Moreover, high percentages of genes that were up- or downregulated 6 h following exercise also demonstrated significantly inversed DNA methylation patterns across one or more CpG sites 3 h following exercise (65% and 82%, respectively). While 30 Fail decreased DNA methylation across various promoter regions versus 80 Fail, transcriptome-wide mRNA and bioinformatics indicated that gene expression signatures were largely similar between bouts. Bioinformatics overlay of DNA methylation and mRNA expression data indicated that genes related to “Focal adhesion,” “MAPK signaling,” and “PI3K-Akt signaling” were significantly affected at the 3 h and 6 h time points, and again this was regardless of bout. In conclusion, extensive molecular profiling suggests that post-exercise alterations in the skeletal muscle DNA methylome and mRNA transcriptome elicited by LL and HL training bouts to failure are largely similar, and this could be related to equal volumes performed between bouts.

## 1. Introduction

Resistance training increases myofiber hypertrophy, whole-tissue hypertrophy, and strength [1]. Training with higher loads (e.g., ~80%+ of a person’s one repetition maximum, or 1RM) and lower volumes (e.g., ~8–12 repetitions per set) generally increases force production capabilities versus lower load training with higher volumes (e.g., 20+ repetitions per set with 30–60% 1RM) [2,3,4,5], although equivocal evidence exists [6,7]. Notwithstanding, most studies to date support that a wide range of loads ≥30 1RM can promote a similar magnitude of skeletal muscle hypertrophy if training is performed to failure [5,6,7,8,9,10,11,12,13].

Although the effects of volume-load manipulations on strength and hypertrophy have been extensively investigated over recent years, much less attention has been given to the potential divergent molecular responses that may occur between higher load and lower load training. Initial findings by Mitchell et al. [5] and Haun et al. [14] suggested that one bout of lower load and higher load failure training similarly elevated post-exercise anabolic signaling events in skeletal muscle (i.e., mTORC1 phosphorylation markers). However, more recent research provides evidence suggesting that different muscle-molecular adaptations may occur between training paradigms. For instance, Lim and colleagues [10] reported that 10 weeks of 30% 1RM training to failure affected markers associated with mitochondrial biogenesis and remodeling versus 80% 1RM training to failure. Haun et al. [15] reported that a six-week lower load and higher volume resistance training program in previously trained men elicited a significant upregulation in sarcoplasmic proteins associated with glycolysis measured via proteomics. In a separate cohort of previously trained men, Vann et al. [16] reported that a 10-week higher load and moderate-to-low volume training program did not alter the sarcoplasmic proteome or promote the glycolytic protein adaptations observed by Haun and colleagues. Vann et al. [4] subsequently examined how six weeks of unilateral lower load versus higher load unilateral leg resistance training affected select molecular outcomes in a third cohort of previously trained men. While shotgun proteomics were not performed, these authors reported that six-week integrated non-myofibrillar protein synthesis rates were significantly greater in the lower load versus higher load trained leg. This collective evidence has led our laboratory to hypothesize that lower load and higher volume resistance training promotes metabolic adaptations relative to higher load training [17].

Unique molecular signaling events induced by exercise training promote alterations in mRNA expression [18], and several lines of evidence suggest that epigenetic modifications to DNA is a mechanism involved in this process [19,20,21]. DNA methylation is one of the most studied epigenetic mechanisms in humans, and recent research supports that a variety of exercise modes, including resistance training, can alter DNA methylation status across various gene promoters [22,23,24]. In mammalian species, DNA methylation primarily occurs at cytosine and guanine dinucleotide-rich sites (CpGs). DNA methylation is catalyzed by DNA methyltransferase (DNMT) enzymes, and DNA de-methylation is catalyzed by ten-eleven translocation methylcytosine dioxygenases (TETs) [20,25]. Increased metabolite flux through glycolysis and the citric acid cycle are thought to alter TET activity [26], and affect the pool of methyl groups available for donation during the methylation process [25]. Current dogma indicates that decreased methylation (hypomethylation) of CpG sites, particularly if occurring in promoter regions, contributes to increased gene expression whereas increased methylation (hypermethylation) contributes to gene silencing [19,21].

It is plausible that divergent genome-wide DNA methylation and mRNA expression signatures induced by higher and lower load resistance training could contribute to some of the unique muscle-molecular phenotypes that have been previously observed between these paradigms. However, neither phenomenon has been previously investigated. Therefore, the purpose of this study was to examine how an acute bout of higher versus lower load resistance exercise to failure affected genome-wide DNA methylation and mRNA transcription profiles in skeletal muscle. Moreover, we sought to determine whether DNA methylation events after each mode of training overlapped with mRNA responses. Herein, lower load and higher load resistance exercise to failure utilized loads that were 30% 1-RM (30 Fail) and 80% 1-RM (80 Fail), respectively. Given previous findings, we hypothesized that 30 Fail training would elicit greater DNA hypomethylation relative to 80 Fail training, and this would correspond to an increased expression of mRNAs associated with mitochondrial biogenesis, mitochondrial remodeling, and glycolysis. 

## 2. Methods 

### 2.1. Ethical Approval and Pre-Screening

This study was conducted with prior review and approval from the Auburn Institutional Review Board (IRB approval #: 20-081 MR 2003), and in accordance with the most recent revisions of the Declaration of Helsinki except not being pre-registered as a clinical trial. The participants recruited for this study were males from the local community that met the following criteria: (i) 18–35 years old, (ii) body mass index ([BMI] body mass in kilograms/ height in meters^2^) not exceeding 35 kg/m^2^, (iii) no known cardio-metabolic disease (e.g., obesity, diabetes, hypertension, heart disease) or any condition contraindicating participation in resistance training or donating muscle biopsies, (iv) have participated in lower-body training at least once per week over the last six months, (v) have a self-reported barbell back-squat one repetition maximum of ≥ 1.5 times body mass. Following verbal and written consent, participants proceeded with study procedures described below.

### 2.2. Study Design

**Figure 1:**
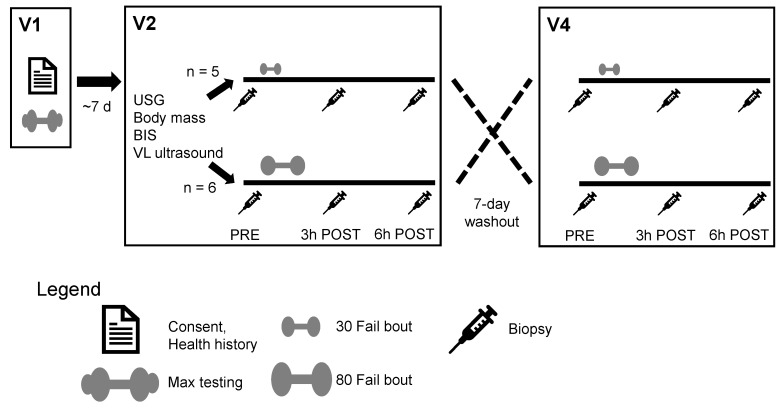
Study design. This schematic represents the timeline for participant consent, max testing, PRE- phenotype testing, experimental resistance exercise bouts, cross-over washout period, and time points for all biopsies. Abbreviations: 30 Fail, 30% one repetition maximum to failure; 80 Fail; 80% one repetition maximum; BIS, bioelectrical impedance spectroscopy (for body composition); USG, urine-specific gravity testing; V1/V2/V4, Visits 1/2/4; VL, vastus lateralis.

Figure 1 provides a schematic of the study design. Participants completed a within-subject design that included a total of five visits to the laboratory. At Visit 1, an informed consent and health history questionnaire were completed. This was followed by barbell back squat and knee extension strength tests as well as body composition testing for phenotyping purposes. At Visit 2 (~7 days following Visit 1), participants completed a battery of baseline (PRE) tests that included measures of height, weight, and vastus lateralis (VL) thickness. Participants also donated a muscle biopsy from the VL prior to the completion of a single bout of resistance exercise using a randomly assigned experimental load (i.e., 30% or 80% estimated 1RM). At both 3 h (3 h POST) and 6 h (6 h POST) following the exercise bout, participants donated additional muscle biopsies from the VL. Seven days following Visit 2, participants completed another bout of resistance exercise using the experimental load not completed at Visit 2 (i.e., if Visit 2 was lifting to failure at 30% 1RM, then Visit 4 implemented 80% 1RM loads). As with Visit 2, VL biopsies were obtained at PRE, 3 h POST, and 6 h POST. Visits 3 and 5 were follow-up visits to ensure biopsy sites were properly healing. During each visit, participants were reminded not to alter their typical diets given that various micronutrients (e.g., B vitamins) can alter methylation status. However, food records were not collected during the intervention. The paragraphs below provide more expanded details on testing procedures.

### 2.3. Testing Sessions

#### 2.3.1. Estimated 1-RM Testing

During Visit 1, participants completed testing to determine their estimated 1-RM (est. 1-RM) on the barbell back squat and leg extension exercises. Testing procedures conformed to the National Strength and Conditioning Association (NSCA) guidelines for strength testing and were performed by Certified Strength and Conditioning Specialists (CSCS). Testing was performed as follows: (i) participants completed a general warm-up of 25 jumping jacks and 10 body weight squats, (ii) for a specific warm-up, 50% of participants’ most recent, self-reported back squat max was executed for 10 repetitions, (iii) additional warm-up reps were completed at approximately 60% (8–10 repetitions), 70% (5–8 repetitions), 80% (3–5 repetitions), 90% (1–3 repetitions) and 95% (1 repetition) of their self-reported 1-RM. Thereafter, 3-RMs for each exercise were assessed within six attempts, and NSCA guidelines were used to convert the 3-RM loads to est. 1-RM values [27].

#### 2.3.2. Hydration Testing

Whole-body hydration was assessed through urine specific gravity (USG) analysis. During Visits 2 and 4, participants donated a urine sample (~5 mL) that was analyzed using a handheld refractometer (ATAGO; Bellevue, WA, USA). All participants had a USG level ≤ 1.020, and this was used as a threshold of sufficient hydration to continue testing [28]. 

#### 2.3.3. Body Composition Testing

During Visit 2 only, height and body mass were measured using a digital column scale (Seca 769; Hanover, MD, USA). Height was measured to the closest 0.5 cm and body mass to the closest 0.1 kg. Bioelectrical impedance spectroscopy (SFB7; ImpediMed Limited, Queensland, AU) was used to estimate body composition for phenotyping purposes. The procedure was performed according to manufacturer’s instructions. The technique was also explained in more depth by Esco et al. [29] and Moon et al. [30]. After participants rested in a supine position for 5 min, two electrodes were placed above and below the right wrist (5 cm apart) and two were placed above and below the anterior portion of the ankle (5 cm apart). Impedance signals were then translated by the device and converted to fat-free mass and fat mass. 

#### 2.3.4. Vastus Lateralis Thickness Assessments

During Visit 2 only, the ultrasound was used to measure VL muscle thickness as reported in Sexton et al. [31] for phenotyping purposes. Participants were instructed to lie supine, with knee and hip fully extended, for a minimum of 5 min before image acquisition. Real-time B-mode ultrasonography (NextGen LOGIQe R8, GE Healthcare, Chicago, IL, USA) and a multi-frequency linear-array transducer (L4-12T, 4–12 MHz, GE Healthcare) was utilized to capture a VL image the transverse plane at approximately 50% the distance between the mid-inguinal crease and the proximal patella. The image depth was adjusted until the edge of the femur was in view, which allowed for a full-thickness view of the VL. All ultrasound images were obtained using a generous amount of water-soluble transmission gel. Associated software (LOGIQe R10.0.4; GE Healthcare) was used to quantify VL thickness, which was defined as the superficial aponeurosis to the deep aponeurosis. All ultrasound images were taken and analyzed by the same investigator.

#### 2.3.5. Skeletal Muscle Biopsy and Processing

During Visits 2 and 4, VL biopsies were collected at PRE, 3 h POST, and 6 h POST using a 5-gauge biopsy needle and local anesthesia. Approximately 50–80 mg of tissue was collected in total. A portion of tissue (~20 mg) was adhered to a cork block via tragacanth gum and preserved in an insulating layer of optimum cutting temperature (OCT) media that was frozen in liquid nitrogen-cooled 2-methylbutane, then stored −80 °C to be later used for histology. Remaining tissue was wrapped in prelabeled foils, flash frozen in liquid nitrogen, and stored at −80 °C for later use. In total, tissue processing occurred within a 5-minute window.

### 2.4. Resistance Exercise Bouts

During Visit 2, participants completed a resistance exercise bout in the morning hours (between 7:00 AM–10:00 AM) following an overnight fast. The bout format was randomly assigned whereby participants performed 4 sets of the back squat and leg extension exercises at either 80% (80 Fail) or 30% (30 Fail) of their est. 1-RM until volitional failure. At the beginning of the bout participants completed a general warm-up of 25 jumping jacks and 10 body weighted squats, followed by a specific warm-up of 10 repetitions at 30–50% and 5–8 repetitions at 75% of the experimental load assigned to them (i.e., 80% or 30% of est. 1-RM). Participants then initiated sets to failure and were provided 5 min of rest between sets. Additionally, participants were given 5 min of rest between squats and knee extensions. Failure was determined as either: (i) an inability to complete a full repetition, (ii) technical errors that potentially compromised safety (e.g., difficulty keeping balance or failure to maintain appropriate posture), or (iii) the participant spent longer than 4 s between repetitions. Participants reporting nausea, malaise, or light-headedness were provided a cereal bar (calories: 170, total fat: 8 g, carbohydrates: 20 g, protein: 4 g) and a sports drink (calories: 80, carbohydrates: 21 g) to reduce symptoms. After the exercise bout was completed, participants were instructed to return to the laboratory 3 h and 6 h later for post-exercise biopsies. Participants were also instructed to not perform rigorous exercise during this time frame.

Seven days later, participants reported back to the laboratory within a two-hour time window of their Visit 2 session and performed the bout format that was not performed the week prior. The warm-up, lifting to failure and between-set recovery parameters, and biopsy sampling times were kept identical to the week prior. Additionally, those that were given post-exercise nutrition the week prior were then given the same items following the second exercise bout.

### 2.5. Biochemical Laboratory Assays

#### 2.5.1. DNA and RNA Isolation

Skeletal muscle samples were retrieved from −80 °C, crushed on a liquid nitrogen cooled stage, weighed to ~10 mg using a laboratory scale (Mettler-Toledo; Columbus, OH, USA), and added to 500 μL of Trizol in 1.7 mL polypropylene tubes. Muscle was homogenized in Trizol (VWR; Radnor, PA, USA) with tight fitting plastic pestles for approximately 30 s and stored in −80 °C overnight to increase RNA yield. The following day samples were removed from −80 °C and allowed to thaw completely at room temperature. Bromochloropropane (100 μL) was added to samples, samples were shaken for 15 s, samples were incubated at room temperature for 2 min, and samples were centrifuged at 12,000 g for 15 min. Once removed from the centrifuge, samples were kept on ice for the remainder of the procedures. Centrifugation yielded three distinct phases including the top aqueous phase containing RNA, the center meniscus containing DNA, and the bottom phase containing protein. Approximately 200 μL of the aqueous phase was removed and placed in a new tube with 500 μL of 100% isopropanol, and the RNA was subsequently pelleted via centrifugation and reconstituted with DEPC water. Reconstituted RNA was then stored at −80 °C and shipped to a commercial laboratory for transcriptome-wide analysis described below.

The remaining DNA and protein were separated by first adding 300 μL of 100% ethanol. Samples were then shaken for 15 s, incubated for 3 min at room temperature, and subsequently centrifuged at 5000 g for 10 min at room temperature. This produced a DNA pellet and a protein supernatant. The supernatant (protein-Trizol-ethanol mixture) was removed, and the DNA pellet was stored at −80 °C until purification was performed using a commercial kit (DNeasy Blood and Tissue Kit, catalog #69504; Qiagen; Germantown, MD, USA) according to manufacturer’s instructions with a minor modification to the elution step. DNA quantity and quality was assessed in duplicate using an absorbance of 260/280 nm provided by a desktop spectrophotometer (NanoDrop Lite; Thermo Scientific; Waltham, MA, USA). DNA was then stored at −80 °C until being shipped on dry ice to a commercial laboratory (TruDiagnostic; Lexington, KY, USA) for the bisulfite conversion and methylation analysis described below.

Transcriptome-wide mRNA analysis. RNA was shipped on dry ice to a commercial laboratory (North American Genomics; Decatur, GA, USA) for transcriptomic analysis using the Clariom S Assay Human mRNA array. Raw data were received as .CEL files and analyzed using the Transcriptome Analysis Console v4.0.2 (Thermo Scientific). More details on the statistical analysis are found later in the Methods section.

#### 2.5.2. DNA Bisulfite Conversion and Methylome Analysis

Bisulfite conversion was performed using an Infinium HD Methylation Assay bisulfite conversion kit (EZ DNA Methylation Kit, Zymo Research, CA, USA). Bisulfite Converted DNA (BCD) was stored at −80 °C until DNA methylation analysis. The Infinium MethylationEpic BeadChip Array (Illumina; San Diego, CA, USA) was performed per the manufacturer’s instructions, and BeadChips were imaged using the Illumina iScan^®^ System (Illumina).

#### 2.5.3. Nuclear Protein Isolations

Nuclear protein isolations from frozen muscle tissue were performed using a commercial kit (Nuclear Extraction Kit, catalog# ab113474; Abcam; Waltham, MA, USA). Briefly, skeletal muscle was retrieved from −80 °C, crushed on a liquid nitrogen cooled stage, and ~15–20 mg of tissue was added to kit pre-extraction buffer containing DTT. The tissue was then homogenized using tight-fitting pestles, incubated on ice for 15 min, and centrifuged for 10 min at 12,000 rpm at 4 °C. The supernatant was removed, 1x pre-extraction buffer containing DTT and protease inhibitors was added to the pellet, and a 15 min incubation ensued with 5 s vortexes every 3 min. The suspension was centrifuged for 10 min at 14,000 rpm at 4 °C. The supernatant was transferred to a new tube and protein concentrations were assessed using a bicinchoninic acid (BCA) assay kit (Thermo Fisher Scientific; Waltham, MA, USA) and microplate spectrophotometer (Synergy H1; Biotek; Winooski, VT, USA). 

#### 2.5.4. Global DNMT and TET Activity Assays

A DNMT activity assay was performed on nuclear extracts using a commercial assay (Colorimetric DNMT Activity Quantification Kit, catalog# ab113467; Abcam) and microplate spectrophotometer (Synergy H1; Biotek). DNMT activity was expressed as absorbance units per microgram of nuclear protein. The average coefficient of variation for duplicate absorbance values was 4.2%. A TET activity assay was also performed on nuclear extracts using a fluorometric commercial assay (TET Hydroxylase Activity Quantification Kit, catalog# ab156913; Abcam) and microplate fluorometer (Synergy H1; Biotek). TET activity was expressed as fluorescence units per microgram nuclear protein. The average coefficient of variation for duplicate fluorescence values was 7.7%.

#### 2.5.5. Immunohistochemistry

Muscle samples that were collected and preserved in OCT as described above were sectioned at a thickness of 10 μm in a cooled (−22 °C) cryostat (Leica Biosystems; Buffalo Grove, IL, USA) and electrostatically adhered to positively charged histology slides. The slides were stored at −80 °C until immunohistochemical staining whereby were removed from the −80 °C, allowed to equilibrate and dry at room temperature for 1.5 h. Sections were then outlined using a hydrophobic pen to retain solutions for the following incubation steps. First, phosphate buffered saline (PBS) was applied for 10 min to rehydrate muscle sections. PBS was then removed, and a 3% peroxide solution was added to sections for 15 min. The peroxide solution was removed, and the slides were washed in PBS for three 5-minute washes on a rocker. Sections were then incubated with TrueBlack Lipofuscin Autofluorescence Quencher solution (biotium; Fremont, CA, USA) for 1 min, and slides were washed in PBS for three 5-minute washes thereafter. The sections were then blocked with a 5% normal goat serum and 2.5% normal horse serum for 1 h and washed in PBS for 5 min. Next, the primary antibody solution was applied (1:20 Mandra, 1:20 of BA_D5, 9:20 and 9:20 5% normal horse serum, all diluted in PBS; all antibodies from Developmental Studies Hybridoma Bank; Iowa City, IA, USA), and slides were incubated overnight in 4 °C. The following day, slides were washed four times in PBS (5 min per wash) A secondary antibody solution was applied for 1 h (1:100 goat anti-mouse IgG1 594 and 1:100 goat anti-mouse IgG2B 488 diluted in PBS). Slides were then washed four times in PBS (5 min per wash) and a DAPI fluorescent dye (1:10,000 DAPI, diluted in deionized water) was then applied for 15 min. Thereafter two 5-minute PBS washes were applied to slides, a 1:1 PBS-glycerol solution was applied around the sections, and glass coverslips were applied thereafter. Immediately following mounting, digital images were captured using a fluorescent microscope (Nikon Instruments, Melville, NY, USA) and a 10× objective. Standardized measurements of type I and type II fiber cross-sectional areas (fCSA) were performed using open-sourced software (MyoVision 2.0) [32,33]. A pixel conversion ratio value of 0.964 µm/pixel was applied to account for the size and bit-depth of images, and a detection range of detection from 500 to 12,000 μm^2^ was used to ensure artifact was removed (i.e., large myofibers which may have not been in transverse orientation, or structures between dystrophin stains which were likely small vessels). Image analysis was also visually inspected to ensure proper analytical fidelity.

### 2.6. Statistical Analyses

Statistical analyses on training repetition/volume and nuclear activity assays were performed using SPSS v26.0 (IBM Corp, Armonk, NY, USA). Prior to analyses, Shapiro–Wilk tests of normality were performed. Two-way ANOVAs were used to assess main effects and potential interactions of condition (30 Fail vs. 80 Fail) × time (PRE, 3 h POST, and 6 h POST). For any main effect that violated the assumption of sphericity, a Greenhouse–Geiser correction was applied. Significant interactions were further interrogated using dependent samples t-tests between conditions at each time point and within each condition over time.

#### 2.6.1. DNA Methylation Analysis

The Partek Genomic Suite V.7 (Partek Inc., St. Louis, MO, USA) was used to process the methylation data as described by Maasar et al. [34]. Briefly, an average detection *p*-value was assessed for all samples to ensure values below 0.01 [35], and any probes outside of this range were removed from analysis. Raw signals for the methylated and unmethylated probes were assessed for the difference between average median methylated and average median unmethylated probes to ensure the recommended difference of 0.5 or less [35]. After the data were imported to Partek Genomics Suite, single nucleotide polymorphism (SNP) associated probes and cross-reactive probes, identified in validation studies [36], were removed from analysis. Functional normalization using a noob background correction was performed for normalization [37]. Then, principal component analyses (PCA), density plots, and box and whisker plots were used for quality control (i.e., no samples exceeded 2.2 standard deviations or presented abnormal distributions). Following normalization and quality control analysis, differentially methylated position analysis was performed. β-values were converted into M-values to represent a more valid distribution of data for analysis of differential methylation [38]. Because both treatments were completed by each participant, paired samples t-tests were used to assess 30 Fail versus 80 Fail at PRE, 3 h POST, and 6 h POST, and significant DMPs at PRE were removed as confounding DMPs. An ANOVA was also used to determine the main effects for condition (30 Fail and 80 Fail over time) and for time (PRE vs. 3 h POST, PRE vs. 6 h POST, and 3 h POST vs. 6 h POST). Differentially methylated CpG positions (DMPs) were subjected to a significance threshold of *p* < 0.01. Additionally, an analysis to quantify differentially methylated regions (DMRs) that contained 2 DMPs or more within a short genomic region was performed using the Bioconductor package DMRcate (DOI: 10.18129/B9.bioc.DMRcate). Pathway enrichment analysis of these DMPs was performed using KEGG (Kyoto Encyclopedia of Genes and Genomes) pathways [39,40,41] via Partek Genomics Suite and Partek Pathway software. All DNA methylation data can be accessed on Gene Expression Omnibus [42] (URL: www.ncbi.nlm.nih.gov/geo/; GEO accession number: GSE220928 (uploaded on 14-12-2022)).

#### 2.6.2. Transcriptome Analysis

Following the quantification of gene expression with the Clariom S microarray, raw .CEL files were uploaded into the Transcriptome Analysis Console v4.0.2 (TAC) (Thermo Scientific). The *H. sapiens* genome was used to generate the reference annotations. Two analyses ensued. First, pairwise comparisons were performed to determine mRNAs that were altered from PRE within each bout. A gene target was considered significant if gene expression exceeded a ±1.5-fold-change from PRE and the *p*-value was less than 0.01. Second, two-way repeated measures (2 × 2) ANOVAs, with the eBayes correction factor applied, were performed to detect genes that differed between bouts from PRE to 3 h POST and PRE to 6 h POST. For this statistical analysis, gene expression was considered significant if a fold-change over times between bouts of ±1.5 was exceeded and the interaction *p*-value was less than 0.01. Bioinformatics using gene lists from both analyses was performed using PANTHER v17.0 [43,44]. All mRNA data can be accessed on Gene Expression Omnibus (URL: www.ncbi.nlm.nih.gov/geo/; GEO accession number: GSE220899 (uploaded on 14-12-2022 and will be made public on 1-6-2023)).

#### 2.6.3. Gene List Overlap Using KEGG Pathways

Gene lists that showed significant DNA methylation and mRNA time effects from PRE to 3 h POST, and 6 h POST were entered into KEGG pathway analysis [39,40,41] to examine significantly associated pathways. Thereafter, common pathways that were predicted to be significantly affected from each dataset (*p* < 0.01) were assessed to determine if pathways overlapped.

## 3. Results

### 3.1. Participant Characteristics

Table 1 contains participant characteristics. The male participants that completed the study (*n* = 11) were 23 ± 4 years old with a body mass of 86 ± 12 kg, a height of 180 ± 7 cm, and a BMI of 27 ± 3 kg/m^2^. Resistance training experience (i.e., training age) was 4 ± 3 years and the est. 1RM for the barbell back squat was 143 ± 33 kg (relative to body mass: 1.7 ± 0.3 kg 1RM/ kg body mass). Average vastus lateralis thickness in all participants was 2.99 ± 0.36 cm, and mean fCSA values from biopsied VL tissue averaged 4259 ± 882 μm^2^ (type I fiber percent: 34.6 ± 16.6, type II fiber percent: 65.4 ± 16.6).

### 3.2. Training Volume Differences between the 30 Fail and 80 Fail Bouts

**Figure 2:**
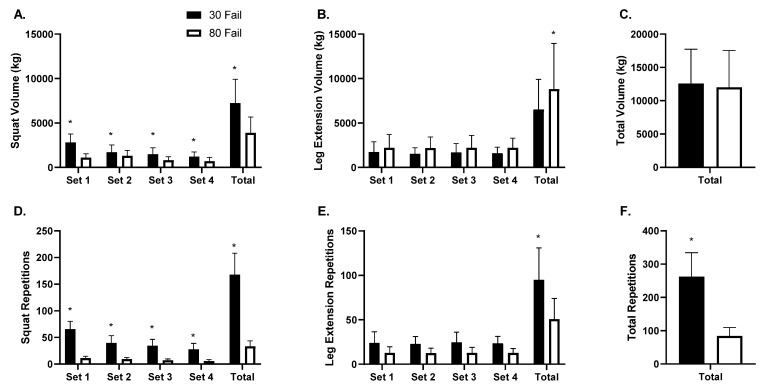
Volume and repetition differences between 30 Fail and 80 Fail bouts. Data are presented as mean ± SD for squat volume across sets and total squat volume (panel **A**), leg extension volume across sets and total leg extension volume (panel **B**), total volume for both exercises (panel **C**), squat repetitions across sets and total squat repetitions (panel **D**), leg extension repetitions across sets and total leg extension repetitions (panel **E**), and total repetitions performed for both exercises (panel **F**). Data are for *n* = 11 participants. Symbol: *, indicates significant difference between conditions per set and in totality (*p* < 0.05). Abbreviations: 30 Fail, 30% one repetition maximum to failure bout; 80 Fail, 80% one repetition maximum to failure bout.

Training volume data are presented in Figure 2 (panels A–C). Barbell back squat training volume (Figure 2A) exhibited a condition by set interaction (*p* < 0.001) and a main effect of condition (*p* < 0.001) whereby more volume was performed in the 30 Fail versus 80 Fail condition. A main effect of set was also evident (*p* < 0.001) whereby average volume decreased across all sets for both conditions. On a per set basis, more back squat volume was performed in the 30 Fail versus the 80 Fail condition (set 1 *p* < 0.01, set 2 *p* = 0.002, set 3 *p* = 0.001, set 4 *p* = 0.005). Moreover, total back squat volume was greater in the 30 Fail versus 80 Fail condition (*p* < 0.001). Leg extension training volume exhibited a main effect of condition (*p* < 0.05, Figure 2B) where more volume was completed in the 80 Fail versus 30 Fail condition. A dependent samples t-test also indicated that significantly more total leg extension volume was completed in the 80 Fail versus 30 Fail condition (*p* = 0.030). However, total lower body training volume between bouts was not significantly different (*p* = 0.570, Figure 2C). 

Training repetition data are presented in Figure 2 (panels D–F). The number of back squat repetitions performed exhibited a condition by set interaction (*p* < 0.001, Figure 2D) and a main effect of condition (*p* < 0.001) whereby more repetitions were performed during the 30 Fail versus 80 Fail condition. Additionally, a main effect of set was evident (*p* < 0.001) whereby repetitions per set decreased in both conditions. Dependent samples t-tests indicated there were significantly more back squat repetitions completed in the 30 Fail versus 80 Fail condition during each set (*p* < 0.001 for sets 1–4) and for all sets combined (*p* < 0.001). The repetitions performed across sets during leg extensions showed a main effect of condition (*p* < 0.001; Figure 2E) whereby significantly more repetitions were completed per set in the 30 Fail versus 80 Fail condition. Total lower body repetitions completed was also significantly greater in the 30 Fail versus 80 Fail bout (*p* < 0.001, Figure 2F).

### 3.3. DNA Methylation Changes with the 30 Fail and 80 Fail Bouts

**Figure 3:**
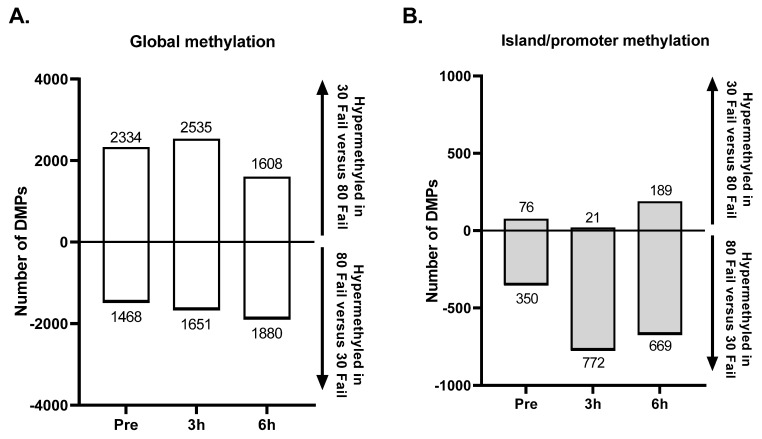
Global and island/promoter methylation differences between bouts. Data are presented as total number of significant differentially methylated positions (DMPs) in 30 Fail compared to 80 Fail (panel **A**), and DMPs present in islands and promoters (panel **B**). Data are for *n* = 11 participants and significance was established as *p* < 0.01 between conditions at each time point. Abbreviations: 30 Fail, 30% one repetition maximum to failure bout; 80 Fail, 80% one repetition maximum to failure bout.

Figure 3 presents differentially methylated position (DMP) and region (DMR) data for significant targets (*p* < 0.01), and these data are presented wherein positive values indicate increased methylation in the 30 Fail versus 80 Fail condition and negative values indicate decreased methylation in the 30 Fail versus 80 Fail condition. There were 3958 significant DMPs between conditions at baseline (*p* < 0.01). Of these 3958 DMPs, only 156 possessed a differentially methylated status at 3 h POST and/or 6 h POST. Therefore, these 156 DMPs were removed from analyses to ensure that changes in each condition were due to the exercise stimuli and not due to altered variation at baseline. 

At PRE (following confounding target removal) there were 3802 DMPs (first bar in Figure 3A), with 1468 DMPs (38.6%) being hypermethylated in 30 Fail versus 80 Fail and 1651 CpGs (61.4%) being hypomethylated in 30 Fail versus 80 Fail. Of these 3802 DMPs, 426 DMPs (11.2%) were in a CpG island within a promoter region (first bar in Figure 3B), and of these 426 DMPs, 76 CpGs (17.8%) were hypermethylated in 30 Fail versus 80 Fail and 772 CpGs (82.2%) were hypomethylated in 30 Fail versus 80 Fail.

At 3 h POST there were 4186 DMPs (middle bar in Figure 3A), with 2535 DMPs (60.6%) being hypermethylated in 30 Fail versus 80 Fail and 1651 CpGs (39.4%) being hypomethylated in 30 Fail versus 80 Fail. Of these 4186 DMPs, 793 DMPs (18.9%) were in a CpG island within a promoter region (middle bar in Figure 3B), and of these 793 DMPs, 21 CpGs (2.6%) were hypermethylated in 30 Fail versus 80 Fail and 772 CpGs (97.4%) were hypomethylated in 30 Fail versus 80 Fail. 

At 6 h POST there were 3488 DMPs (third bar in Figure 3A), with 1608 DMPs (46.1%) being hypermethylated in 30 Fail versus 80 Fail and 1880 DMPs (53.9%) being hypomethylated in 30 Fail versus 80 Fail. Of these 3488 DMPs, 858 DMPs (24.6%) resided in CpG islands within a promoter region (third bar in Figure 3B), and of these, 189 were hypermethylated in 30 Fail versus 80 Fail (22%) and 669 were hypomethylated in 30 Fail versus 80 Fail (88%).

Regarding DMRs at PRE between the 30 Fail and 80 Fail bouts, there were 68 DMRs that all contained 2 DMPs (*p* < 0.01). Regarding DMRs at 3 h POST between the 30 Fail and 80 Fail bouts, there were one hundred fifty-five DMRs with two or more significant DMPs (*p* < 0.01), nine of these DMRs contained three DMPs (associated genes: DIP2B, AHCYL, TMEM134, FAM216A, SLC8B1, SERF2, TOM1L2, TEX14, and TXN2), and only one DMR contained four DMPs (associated gene: EIF4B). Further, forty-nine of these one hundred fifty-five DMRs (32%) were in CpG islands within promoter regions, with two genes containing three DMPs (associated genes: FAM216 and TOM1L2) and one gene containing four DMPs (associated gene: EIF4B).

At 6 h POST between the 30 Fail and 80 Fail bouts there were sixty-seven DMRs with at least two significant DMPs. Three DMRs contained three DMPs (associated genes: SIN3A, RBM39, and PEG10), and only one DMR contained four DMPs (associated gene: MAP3K3). Of these sixty-seven DMRs, sixty-five DMRs (97%) were in CpG islands within promoter regions, with these DMRs containing three or more DMPs.

### 3.4. Nuclear TET and DNMT Activity

**Figure 4:**
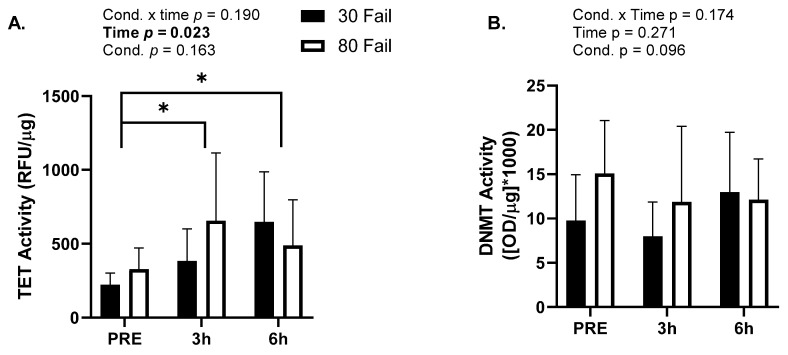
Nuclear TET and DNMT activities between bouts. All data are presented as mean ± SD for global TET activity (*n* = 10) from PRE to 3 h POST and 6 h POST resistance exercise (panel **A**) and DNMT activity (*n* = 9) from PRE to 3 h POST and 6 h POST resistance exercise (panel **B**); Symbol: *, indicates a significant change from PRE regardless of bout. Abbreviations: 30 Fail, 30% one repetition maximum to failure bout; 80 Fail, 80% one repetition maximum to failure bout; DNMT, DNA Methyltransferase; OD, optical density; RFU, relative fluorescent units; TET, Ten-Eleven Translocase.

Given robust alterations in DNA methylation with both training bouts, we opted to investigate global TET (demethylating) and DMNT (methylating) activities from nuclear lysates. Due to tissue limitations, *n* = 10 participants yielded enough nuclear lysate material to perform the TET activity assay and *n* = 9 participants yielded enough nuclear lysate material to perform the DNMT activity assay. There was a significant main effect of time for TET activity (*p* = 0.023, Figure 4A) where average TET activity increased at both post-exercise time points. However, there was no significant main effect of condition (*p* = 0.163) or condition by time interaction (*p* = 0.190). For DNMT activity there were no significant main effects of time (*p* = 0.271), condition (*p* = 0.096), or condition by time interaction (*p* = 0.174; Figure 4B).

### 3.5. mRNA Expression Data and Bioinformatics

There were 21,488 genes probed to identify differentially expressed genes (±1.5-fold, *p* < 0.01; termed ‘DEGs’). There were 889 significant DEGs at PRE between conditions (*p* < 0.05); thus, these DEGs were removed from analyses to ensure that changes in each condition were due to the exercise stimuli and not due to altered variation at baseline.

**Figure 5:**
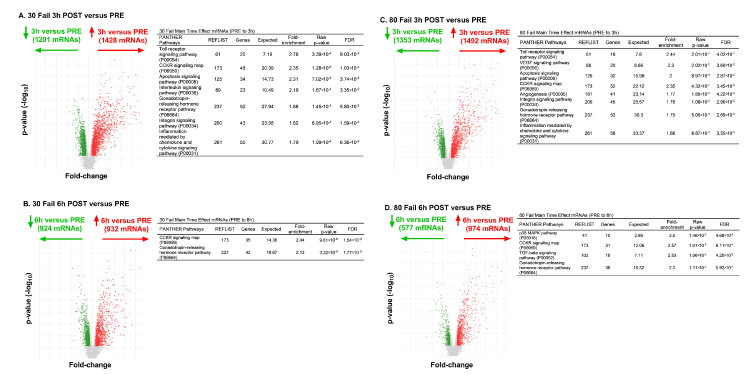
Time effect mRNAs between bouts with pathway enrichment data. Differentially expressed genes (DEGs) within the 30 Fail and 80 Fail bouts viewed as volcano plots and PANTHER pathway enrichment. More specifically, 30 Fail training DEGs from PRE to 3 h POST (panel **A**), 30 Fail training DEGs from PRE to 6 h POST (panel **B**), DEGs from PRE to 3 h POST with 80 Fail training (panel **C**), and DEGs from PRE to 3 h POST with 80 Fail training (panel **D**). Data are for *n* = 11 participants, all DEGs have a fold-change from PRE of ±1.5 (*p* < 0.01), and pathway analysis was significant if False Discovery Rate (FDR) *p* < 0.05. Abbreviations: 30 Fail, 30% one repetition maximum to failure bout; 80 Fail, 80% one repetition maximum to failure bout; REFLIST, reference gene list across human genome.

From PRE to 3 h POST with 30 Fail training, 1428 mRNAs were upregulated and 1201 mRNAs were downregulated (Figure 5A). Bioinformatics indicated that mRNAs in the following pathways were significantly enriched (*p* < 0.001, FDR < 0.05): (i) Toll receptor signaling (fold-enrichment = 2.78), (ii) CCKR signaling (fold-enrichment 2.35), (iii) apoptosis signaling (fold-enrichment = 2.31), (iv) interleukin signaling (fold-enrichment = 2.19), (v) gonadotropin-releasing hormone receptor signaling (fold-enrichment = 1.86), (vi) integrin signaling (fold-enrichment = 1.82), (vii) inflammation mediated by chemokine and cytokine signaling (fold-enrichment = 1.79). From PRE to 6 h POST with 30 Fail training 932 mRNAs were upregulated and 924 mRNAs were downregulated (Figure 5B). Bioinformatics indicated that mRNAs in the following pathways were significantly enriched: (i) CCKR signaling (fold-enrichment 2.44), (ii) gonadotropin-releasing hormone receptor signaling (fold-enrichment = 2.13).

From PRE to 3 h POST with 80 Fail training 1492 mRNAs were upregulated and 1353 mRNAs were downregulated (Figure 5C). Bioinformatics indicated that mRNAs in the following pathways were significantly enriched: (i) Toll receptor signaling (fold-enrichment = 2.78), (ii) VEGF signaling (fold-enrichment = 2.30), (iii) apoptosis signaling (fold-enrichment = 2.00), (iv) CCKR signaling (fold-enrichment = 2.35), (v) angiogenesis (fold-enrichment = 1.77), (vi) integrin signaling (fold-enrichment = 1.76), (vi) gonadotropin-releasing hormone receptor signaling (fold-enrichment = 1.75), (vii) inflammation mediated by chemokine and cytokine signaling (fold-enrichment = 1.68). From PRE to 6 h POST with 80 Fail training, 974 mRNAs were upregulated and 577 mRNAs were downregulated (Figure 5D). Bioinformatics indicated that mRNAs in the following pathways were significantly enriched: (i) p38 MAPK signaling (fold-enrichment 2.44), (ii) CCKR signaling (fold-enrichment = 2.57), (iii) TGF-beta signaling (fold-enrichment = 2.53), (iv) gonadotropin-releasing hormone receptor signaling (fold-enrichment = 2.30).

**Figure 6:**
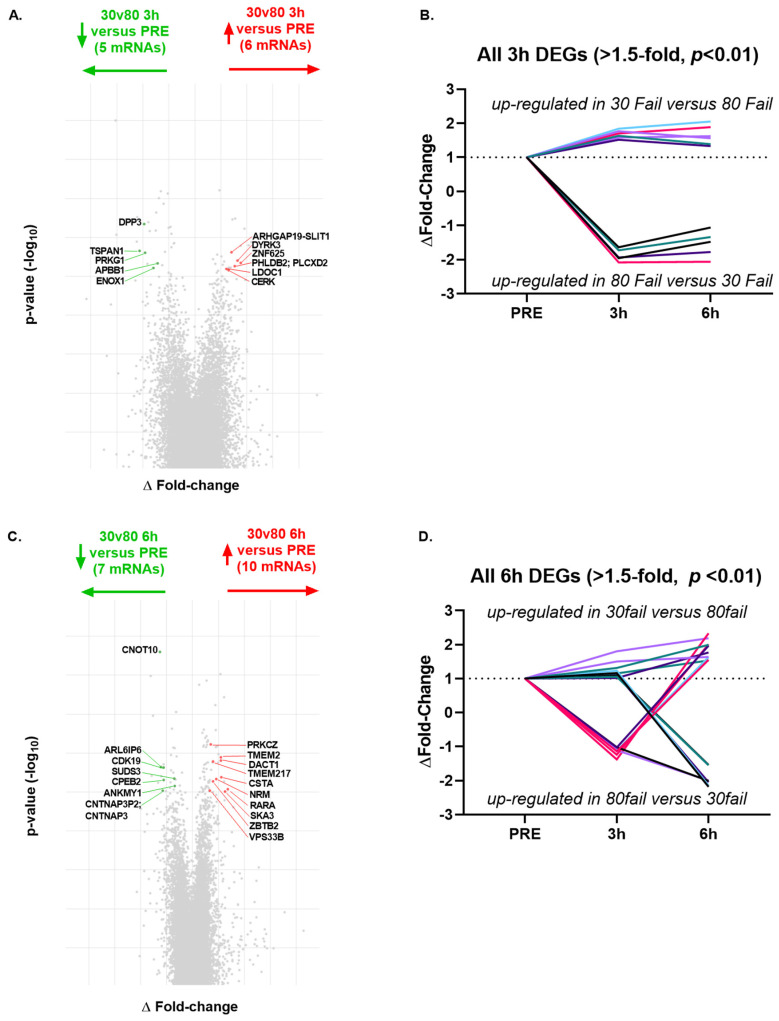
Differentially expressed mRNAs between bouts at both post-exercise time points. All data are significant differentially-expressed genes (DEGs) following 30 Fail versus 80 Fail for the ΔFold-change in gene expression plotted against *p*-value (−log_10_ transformed) at 3 h POST versus PRE (panel **A**), the ΔFold-change over time for significant DEGs at 3 h POST versus PRE (panel **B**), the ΔFold-change in gene expression plotted against *p*-value (−log_10_ transformed) at 6 h POST versus PRE (panel **C**), and the ΔFold-change over time for significant DEGs at 6 h POST versus PRE (panel **D**). Data are for *n* = 11 participants and the significance threshold was established as ±1.5 ΔFold-change, *p* < 0.01. Abbreviations: 30 Fail, 30% one repetition maximum to failure bout; 80 Fail, 80% one repetition maximum to failure bout.

Regarding condition by time interactions (delta-delta fold-change ±1.5, *p* < 0.01), there were only 11 significant DEGs from PRE to 3 h POST (Figure 6A,B), and 17 significant DEGs between bouts from PRE to 6 h POST (Figure 6C,D). Given the low number of differentially expressed genes between bouts over time, no pathways were predicted to differ between bouts at the 3 h POST or 6 h POST time points.

### 3.6. Overlapping Genome-wide Methylome and Transcriptome Results

**Figure 7:**
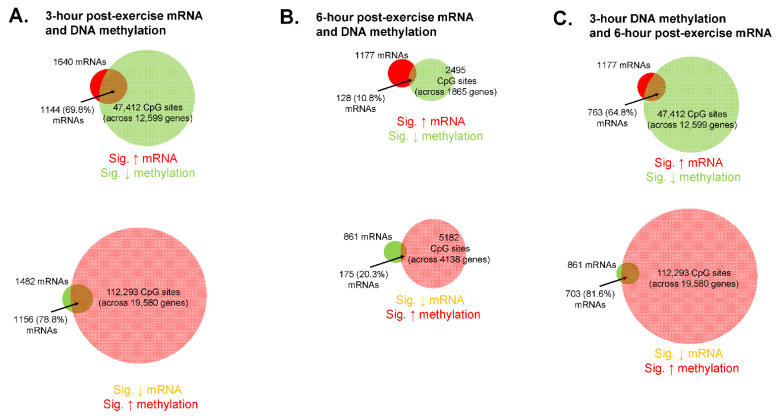
Transcriptome and methylation signatures overlayed. Venn diagrams illustrating mRNAs and DNA methylation values that showed significant main effects of time from PRE where red circles indicate targets that were up-regulated from PRE to 3-/6-h and green circles indicate targets that were down-regulated from PRE to 3-/6-h. Panel **A** contains significantly altered targets from PRE to 3 h post-exercise only. Panel **B** contains significantly altered targets from PRE to 6 h post-exercise only. Panel **C** contains DNA methylation alterations that occurred from PRE to 3 h post-exercise and mRNA expression alterations from PRE to 6 h post-exercise. Data are from *n* = 11 participants, differentially methylated CpG sites were considered significantly altered from PRE if *p* < 0.01, and mRNAs were considered significantly altered from PRE if ±1.5 fold-change (*p* < 0.01).

An overlay of methylome and transcriptome data can be found in Figure 7. Notably, these analyses only considered mRNAs and DNA methylation values that showed significant main effects of time from PRE. 

A relatively high level of DNA methylation events occurred from PRE to 3 h POST, as there was a significant decrease in the methylation status of 69,696 CpG sites and significant increase in 170,255 CpG sites (Figure 7A). Of the 1640 mRNAs that were significantly up-regulated at 3 h POST, 1144 (or 68.8%) of these mRNAs had one or more associated CpG site that was hypomethylated at this time point. Of the 1482 mRNAs that were significantly down-regulated 3 h POST, 1156 (or 78.8%) of these mRNAs had one or more associated CpG site that was hypermethylated at this time point.

Compared to 3 h POST there were fewer DNA methylation events that occurred 6 h POST, as there was a significant decrease in the methylation status of 3578 CpG sites and significant increase in 8841 CpG sites (Figure 7B). Of the 1177 mRNAs that were significantly up-regulated 6 h POST, 128 (or 10.8%) of these mRNAs had one or more associated CpG site that was hypomethylated at this time point. Of the 861 mRNAs that were significantly down-regulated 6 h POST, 175 (or 20.3%) of these mRNAs had one or more associated CpG site that was hypermethylated at this time point.

Because DNA methylation precedes alterations in gene transcription, we also investigated DNA methylation changes at 3 h POST relative to 6 h POST mRNA expression changes (Figure 7C). Of the 1177 mRNAs that were significantly up-regulated at 6 h POST, 763 (or 64.8%) of these mRNAs had one or more associated CpG site that was hypomethylated at 3 h POST. Of the 861 mRNAs that were significantly down-regulated 6 h POST, 703 (or 81.6%) of these mRNAs had one or more associated CpG site that was hypermethylated at 3 h POST.

Finally, Table 2 contains KEGG analysis results of significantly altered pathways predicted to be affected following exercise, regardless of bout, according both the DNA methylation and mRNA expression signatures. When removing disease-related pathways from the 3 h POST analysis (e.g., “Yersina infection,” “Salmonella infection,” “Cushing syndrome,” etc.), 50 KEGG pathways were significantly enriched (*p* < 0.01) according to the DNA methylation data, 22 KEGG pathways were significantly enriched (*p* < 0.01) according to the mRNA expression data, and 14 (64%) of these pathways overlapped. When removing disease-related pathways from the 6 h POST analysis, 40 KEGG pathways were significantly enriched (*p* < 0.01) according to the DNA methylation data, 16 KEGG pathways were significantly enriched (*p* < 0.01) according to the mRNA expression data, and four (25%) of these pathways overlapped. Of the 50 KEGG pathways were significantly enriched (*p* < 0.01) according to the 3 h POST DNA methylation data and 16 KEGG pathways were significantly enriched (*p* < 0.01) according to the 6 h POST mRNA data, 13 (81%) of these pathways overlapped.

While not highlighted in Table 2, it is notable that three pathways were predicted to be significantly altered according to the DNA methylation and mRNA signatures in all 3 h and 6 h post exercise comparisons included “MAPK signaling,” “PI3K-Akt signaling,” and “Focal adhesion.”

## 4. Discussion

Research interest into how exercise affects genome-wide skeletal muscle DNA methylation has increased in recent years, and multiple associated reviews have been published [25,45,46,47]. Barres et al. [19] were the first to report that the hypomethylation of various metabolic genes occurs in skeletal muscle following a single high-intensity aerobic exercise session in humans, and that these events corresponded with an alteration in the mRNA expression levels of these genes. A 2021 study from Maasar and colleagues compared the skeletal muscle DNA methylome responses to two bouts of running exercise [34]. The authors reported that CpG sites associated with several metabolic genes exhibited more robust demethylation responses to the higher intensity bout of change-of-direction running versus straight line running. Telles et al. [48] more recently examined how resistance exercise, high-intensity interval exercise, or the combination of both affected the mRNA expression and DNA methylation of select myogenic regulatory factors (MYOD1, MYF5, and MYF6). All exercise protocols were reported to promote DNA demethylation of these genes 4 h and 8 h post-exercise, and the mRNA expression of MYOD1 and MYF6 were elevated 4 h following exercise. Finally, acute resistance exercise in untrained males has been demonstrated to promote DNA hypomethylation and increased expression of mRNAs associated with matrix/actin structure and remodeling, mechano-transduction, and TGF-beta signaling and protein synthesis [22,24]. While these investigations have been insightful, it is difficult to compare our findings to data from these aforementioned studies given the differences in exercise modes and muscle biopsy sampling time points. The current study adds meaningful insight to this body of literature. First, the lower load (30 Fail) resistance exercise bout promoted a more robust CpG island/promoter hypomethylation response at both post-exercise time points relative to higher load (80 Fail) resistance exercise. Further, a gene related to translation initiation and protein synthesis (EIF4B) possessed four DMRs in the promoter region that were hypomethylated in the 30 Fail versus 80 Fail condition 3 h following exercise. Whether these acute responses eventually translate to differential training adaptations remains to be determined. Further, whether this differential methylation signature in one gene between conditions (EIF4B) led to differences in muscle protein synthesis was not examined. Notwithstanding, and in agreement with the data from Massar and colleagues, this finding supports that implementing different training styles elicits modest but unique post-exercise DNA methylation signatures. 

Another novel implication from these data is that most DNA methylation events occurred 3 h versus 6 h post-exercise regardless of bout (239,951 versus 12,419 CpG sites significantly hyper- or hypomethylated at these respective time points, *p* < 0.01). Indeed, long-lived alterations in skeletal muscle DNA methylation have been reported to occur in response to weeks of resistance training [23], and this phenomenon has been posited to serve as an “epigenetic memory” mechanism to promote more streamlined gene expression responses to subsequent training bouts. However, the data herein also illustrate that a bout of resistance exercise can lead to appreciably rapid and robust genome-wide alterations in skeletal muscle DNA methylation patterns within a 3-h time frame following an exercise bout (and regardless of condition). Accordingly, data in Figure 7 show that the percentage of significantly altered mRNAs that also demonstrated significantly inversed methylation patterns across one or more CpG sites was appreciably higher at 3 h versus 6 h following exercise (~75% versus ~15%, respectively). Moreover, high percentages (65% and 82% respectively) of genes that were up- or downregulated 6 h following exercise also demonstrated significantly inversed DNA methylation patterns across one or more CpG sites 3 h following exercise. Only a handful of studies have sought to compare DNA methylation events to corresponding mRNA expression patterns following one or multiple bouts of resistance exercise. For instance, Laker and colleagues [49] reported that only 2% of differentially expressed genes from a one-week (three-bout) resistance training intervention were also differentially methylated. However, these data were confounded by participants being assigned to a high fat diet and the post-intervention biopsy was collected one day following the last exercise bout. Seaborne and colleagues reported that an acute bout of resistance exercise in eight untrained males led to ~10,000 CpG sites becoming hypomethylated and ~7500 becoming hypermethylated [23]. Interestingly, DNA methylation signatures were not associated with changes in mRNA expression until participants had undergone a chronic training paradigm [23]. Additionally, when acute and chronic methylome and transcriptome data were overlapped, ~40% of differentially expressed genes were shown to be associated with altered DNA methylation signatures [24]. Telles et al. [48] more recently reported that an interrelated, but not time-aligned response, of myogenic regulatory factor gene demethylation and mRNA expression occurred up to 8 h following resistance exercise and other exercise modalities. However, their analyses were only limited to three genes, and it is notable that all these prior studies examined untrained participants. When considering our data in lieu of this prior evidence, it is becoming increasingly evident that resistance exercise-induced alterations in skeletal muscle DNA methylation and mRNA expression patterns are interrelated, albeit this relationship appears to be more coupled rapidly following exercise (i.e., within a 3-hour window) rather than 6+ hours or days following an exercise bout.

Bioinformatics overlay analysis on DNA methylation and mRNA expression changes from PRE (regardless of bout) was also insightful. As seen in Table 2, the three pathways were predicted to be significantly altered whereby genes showed overlap at the DNA methylation and mRNA expression levels 3 h and 6 h following exercise included “MAPK signaling,” “PI3K-Akt signaling,” and “Focal adhesion.” Indeed, several studies examining the acute signaling responses to a bout of resistance exercise have implicated that proteins encoded from genes of these pathways show altered phosphoprotein statuses [50,51,52,53,54]. However, data regarding how resistance exercise affects the DNA methylation status and mRNA responses of genes associated with these pathways are sparse. Seaborne and colleagues utilized KEGG pathway analysis with acute and chronic resistance training to report that the skeletal muscle DNA methylation statuses of genes related to “MAPK signaling” and “Focal adhesion” are significantly altered [22]. Turner et al. [24] have overlapped methylome and pooled transcriptome data to report that chronic resistance training affects genes associated with “matrix/actin structure and remodeling,” “Focal adhesion,” and “TGF-beta signaling and protein synthesis.” Hence, the current data continue to provide evidence that post-exercise alterations in DNA methylation and mRNA expression patterns are predicted to affect select genes associated with these cellular processes. Also notable, the pathways predicted to be affected according to 3 h post-exercise DNA methylation changes and 6 h post-exercise mRNA expression changes showed the greatest overlap (81%) compared to 3 h overlay data (64%) and 6 h overlay data (25%). As has been discussed above, this finding continues to indicate that earlier DNA methylation events better align with mRNA expression events at later time points.

A final novel finding resulting from our nuclear lysate assays is the observed changes in nuclear TET, but not DNMT, activity. Comparing our data to other research findings is not possible since this is the first investigation to determine how nuclear DNMT and TET activities are altered by exercise. Indeed, it is plausible that the exercise-induced increase in TET activity observed herein promoted a reduction in DNA methylation. However, there are limitations to this interpretation. First, we were unable to determine which TET isoforms were operative in affecting global TET activity, and it is also possible that the activities of specific DNMT isoforms were differentially affected to result in a nullified post-exercise global DNMT activity change. Second, data presented in Figure 7 indicate that appreciably more CpG sites showed increased methylation relative to CpG sites showing decreased methylation, which indicates that the activities of one or multiple DNMTs were likely elevated during or within the first 3 h of exercise. Given the novelty and elusiveness of these findings, more refined analytical approaches are needed to determine how exercise affects the activities of various TET and DNMT isoforms. As an aside, there are interesting data linking TET activity to muscle function. In an elegant series of experiments by Wang et al. [55], the authors reported that TET2 knockout mice experienced severe muscle dysfunction. The authors used these data as well as in vitro experiments to conclude that TET2 activity is essential for muscle regeneration as well as myoblast differentiation and fusion. Although the implications of these preclinical data are difficult to relate to the current study, the data from Wang and colleagues reiterate the need to examine nuclear TET activity in human skeletal muscle during periods of exercise training and various disease states.

### Experimental Limitations

The present study possesses limitations including a small sample size, the younger healthy male sample population, and muscle sample collection time points. A larger number of more diverse participants and/or muscle samples being collected at additional time points across a 24 to 72 h time frame may have yielded more insight. Moreover, although participants were instructed to not deviate from their typical diets throughout the intervention, dietary recalls were not obtained to account for how self-reported micronutrient statuses may have affected results. This is an unresolved limitation and, while dietary effects were not the emphasis of the current study, we acknowledge that inter-individual differences in macro- and micronutrient intakes likely played a role in some of the observed methylation outcomes. Finally, our significance thresholds in relation to the DNA methylation data warrant discussion. Indeed, applying a *p* < 0.01 threshold to methylation array data can be interpreted by some as being a rather liberal threshold cutoff; refer to Mansell et al. [56] who suggest a significance threshold of *p* < 9 × 10^−8^ should be considered. However, when applying such stringent thresholds to our current dataset we find, for instance, that (in referral to Figure 3 data) there are no DMPs that differ (*p* < 9 × 10^−8^) between 30 Fail and 80 Fail at PRE, 3 h, or 6 h post-exercise. Importantly, with our approach we demonstrated a degree of overlap between the pathway analysis at the methylation level (using FDR) and the mRNA level, with enrichment in the same pathways of “Focal adhesion,” “MAPK signaling,” and “PI3K-Akt signaling.” These pathways have also been shown to be differentially methylated and differentially methylated and expressed after resistance exercise in independent cohorts [22,24]. Therefore, we surmise that the current findings, whereby methylation data were deemed significant at *p* < 0.01, demonstrate biological relevance given the degree of overlap discovered at both the methylation level and transcriptome level as well as exhibiting agreement with previous findings in the literature. Notwithstanding, we do realize that applying *p* < 0.01 is a liberal approach and want to make the reader aware of this aspect of the study.

## 5. Conclusions

In conclusion, 30 Fail and 80 Fail resistance exercise bouts produced unique DNA methylation responses across various gene promoters, albeit largely similar transcriptomic responses. These data continue to add insightful information to the body of literature comparing the muscle-molecular responses of higher load versus lower load resistance training paradigms.

## Figures and Tables

**Table 1:** Participant characteristics.

Variables	Mean ± SD
Age (years)	23 ± 4
Training Age (years)	4 ± 3
Body Mass (kg)	86 ± 12
Height (cm)	180 ± 7
BMI (kg/m^2^)	27 ± 3
Est. squat 1-RM (kg)	143 ± 33
Squat (kg)/Body Mass (kg)	1.7 ± 0.3
VL thickness (cm)	2.99 ± 0.36
Mean fCSA (µm^2^)	4259 ± 882
Type I Fiber Percent	34.6 ± 16.6
Type II Fiber Percent	65.4 ± 16.6

Data are for the 11 participants and presented as mean ± standard deviation (SD) values. Abbreviations: BMI, body mass index; 1-RM, one repetition maximum; VL, vastus lateralis; fCSA, fiber cross-sectional area. All data were collected prior to first experimental resistance training bout.

**Table 2:** KEGG pathway analysis and overlap of DNA methylation and mRNA expression data.

Pathways that Overlapped	Fold-Enrichment (DNA Meth., mRNA)	% Genes Affected in Pathway (DNA Meth., mRNA)	Pathway *p*-Values (DNA Meth., mRNA)
*3 h post-exercise DNA meth. and mRNA* *			
TNF signaling pathway	6.9, 2.4	97.3, 1.5	0.001, 2.9 × 10^−9^
MAPK signaling pathway	12.6, 1.6	96.3, 2.7	3.3 × 10^−6^, 3.4 × 10^−6^
HIF-1 signaling pathway	5.3, 2.0	96.3, 1.2	0.005, 2.4 × 10^−5^
FOXO signaling pathway	4.7, 1.9	95.4, 1.9	0.007, 6.6 × 10^−5^
TGF-beta signaling pathway	8.6, 2.0	98.9, 1.0	0.0002, 8.9 × 10^−5^
Other KEGG pathways that overlapped at 3 h post-exercise (*n* = 9 additional): Apoptosis, Chemokine signaling pathway, PI3K-Akt signaling pathway, Rap1 signaling pathway, Focal adhesion, Leukocyte transendothelial migration, Hippo signaling pathway, Insulin signaling pathway, C-type lectin receptor signaling pathway			
*6 h post-exercise DNA meth. and mRNA ^†^*			
MAPK signaling pathway	17.9, 1.9	40.1, 2.8	1.7 × 10^−8^, 5.1 × 10^−6^
PI3K-Akt signaling pathway	7.1, 1.5	33.0, 2.7	0.0008, 0.0004
Focal Adhesion	25.0, 1.6	47.5, 1.7	1.4 × 10^−11^, 0.006
Wnt signaling pathway	8.2, 1.6	38.1, 1.6	0.0002, 0.001
*3 h post-exercise DNA meth. and 6 h mRNA* *			
MAPK signaling pathway	12.6, 1.9	96.3, 2.8	3.3 × 10^−6^, 5.1 × 10^−6^
Autophagy	7.7, 2.1	97.1, 1.5	0.004, 1.1 × 10^−4^
TNF signaling pathway	6.9, 2.2	97.3, 1.3	0.001, 1.8 × 10^−4^
HIF-1 signaling pathway	5.3, 2.2	96.3, 1.2	0.005, 3.0 × 10^−4^
Th17 cell differentiation	6.3, 2.0	97.2, 1.1	0.0017, 0.0016
Other KEGG pathways that overlapped using 3 h post-exercise DNA methylation and 6 h post-exercise mRNA data (*n* = 8 additional): Apoptosis, FoxO signaling pathway, TGF-beta signaling pathway, Insulin signaling pathway, PI3K-Akt signaling pathway, Adipocytokine signaling pathway, Focal Adhesion, Wnt signaling pathway			

**Legend:** These data were derived from entering gene lists into the KEGG pathway analysis queue, and gene lists were derived from genes that showed significant (*p* < 0.01) time effects for DNA methylation (DNA meth.) and mRNA expression changes from pre-exercise to 3 h and 6 h following exercise regardless of bout (i.e., those showing significant time effects). Symbols: *, indicates these lists were sorted by lowest *p*-value according to mRNA bioinformatics; ^†^, indicates only 4 pathways overlapped at the 6 h post-exercise time point.

## Data Availability

Queries not related to DNA methylation analysis can be addressed by M.D.R. (mdr0024@auburn.edu). Questions related to DNA methylation can be addressed by A.P.S. (adams@nih.no). All mRNA data can be accessed on Gene Expression Omnibus (URL: www.ncbi.nlm.nih.gov/geo/; GEO accession number: GSE220899 (uploaded on 14-12-2022 and will be made public on 1-6-2023)). All DNA methylation data can also be accessed on GEO (URL: www.ncbi.nlm.nih.gov/geo/; GEO accession number: GSE220928 (uploaded on 14-12-2022).
